# Supplementary material for: Nuclear PTEN safeguards pre-mRNA splicing to link Golgi apparatus for its tumor suppressive role
Source: Nat Commun. 2018 Jun 19;9:2392. doi: 10.1038/s41467-018-04760-1 (PMC6008332; doi:10.1038/s41467-018-04760-1)
Supplement: Supplementary file 1 — Supplementary Information [file 41467_2018_4760_MOESM1_ESM.pdf]

Supplementary Information

Nuclear PTEN safeguards pre-mRNA splicing to link Golgi apparatus for its tumor suppressive role

Shen et al.

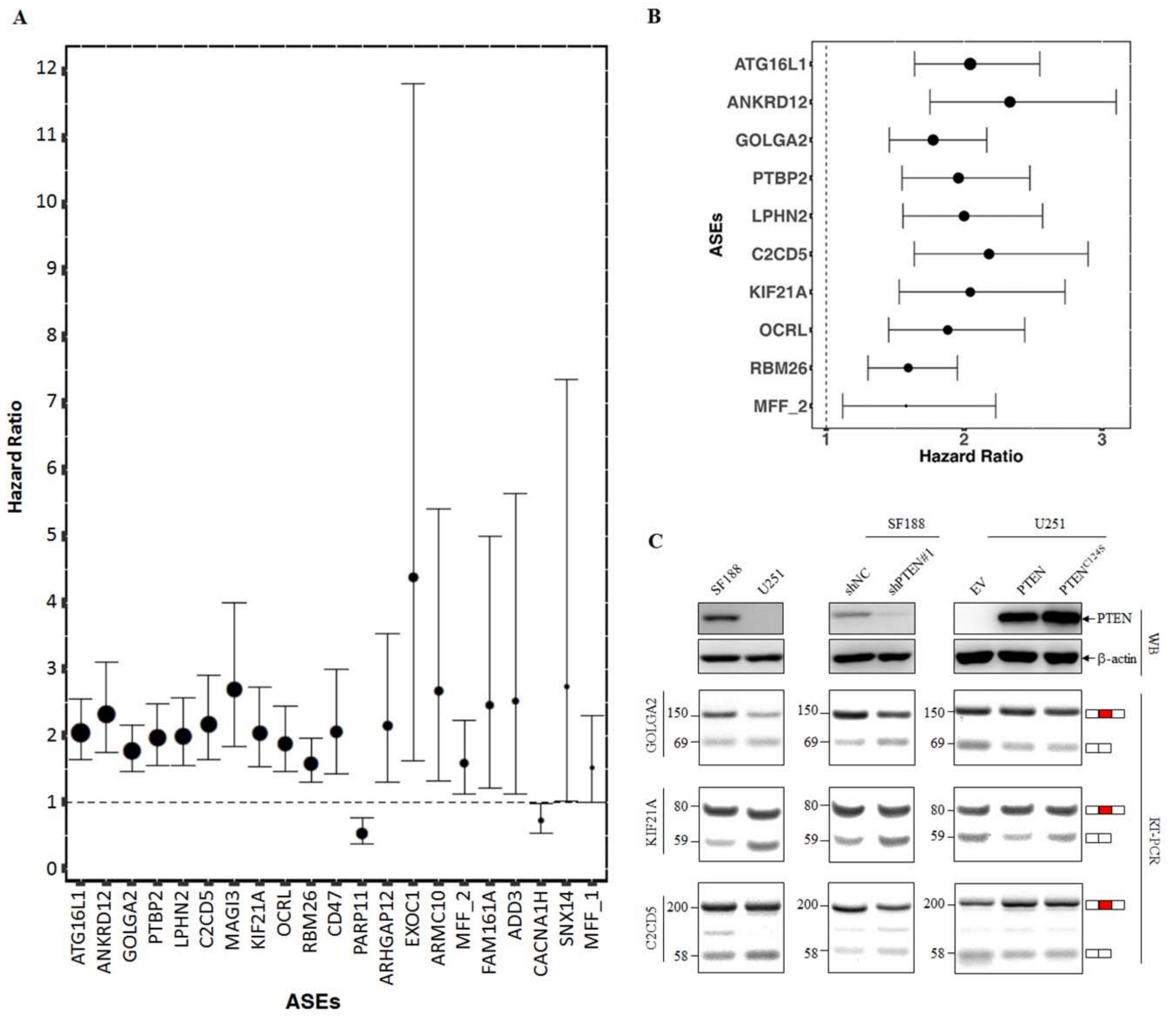

Supplementary Figure 1. PTEN regulates alternative splicing in cancer. (**A**) The hazard ratios of 262 ASEs identified in 293T cells were analyzed in GBMLGG, and ones with significant impact ($P < 0.05$) on patient survival were shown. (**B**) The hazard ratios of the ten cancer-related, PTEN- and survival-correlated ASEs from Fig. 2f in GBMLGG. (**C**) The alternative splicing of GOLGA2, KIF21A and C2CD5 were verified in SF188 compared to U251 cells (left), SF188 with or without PTEN knockdown (middle), and U251 cells overexpressed with EV, PTEN, or $PTEN^{C124S}$ (right).

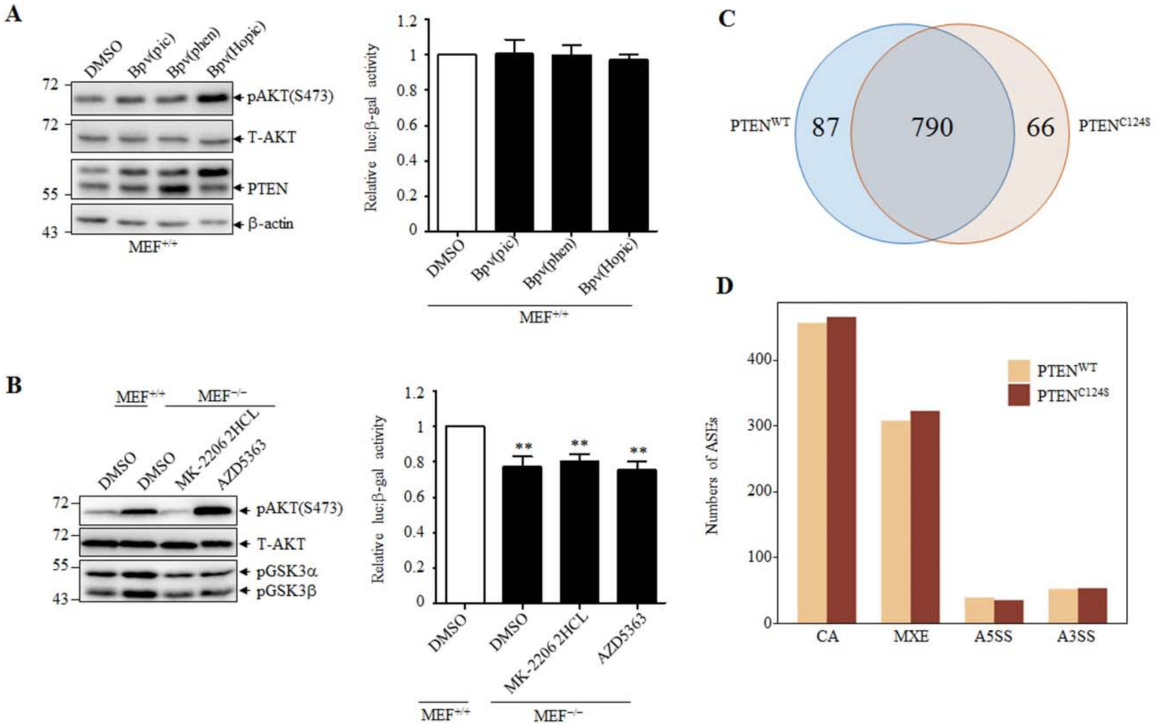

Supplementary Figure 2. PTEN regulates AS in its phosphatase-independent manner. (**A**) PTEN$^{+/+}$ MEF cells transfected with pTN24 were further treated with bisperoxovanadium 2-carboxypyridine (bpV(pic)), bisperoxovanadium 1,10 phenanthroline (bpV(phen)), bisperoxovanadium 5-hydroxypiridine-2-carboxyl (bpV(HOpic)) or DMSO for 12 h. Immunoblotting of proteins as indicated (left) and the ratios of luciferase expression relative to β-galactosidase expression (right) are shown. Data represent means with bar as s.d. of three independent experiments in the right panel. (**B**) MEF cells transfected with pTN24 were treated with MK-2206 2HCL, AZD5363 or DMSO for 12 h. Immunoblotting of proteins as indicated(left) and ratios of luciferase expression relative to β-galactosidase expression (right) are shown. Data represent means with bar as s.d. of three independent experiments. **, $p < 0.01$; two-sided unpaired *t*-test. (**C, D**) PTEN^{WT} or PTEN^{C124S} along with EV were transduced into shPTEN#1-infected 293T cells by lenti-virus. The Venn diagram of shPTEN#1-induced ASEs (C) and numbers of four kinds of shPTEN#1-induced ASEs (D) identified in Fig. 1c rescued by overexpression of PTEN^{WT} or PTEN^{C124S} compared to EV was shown.

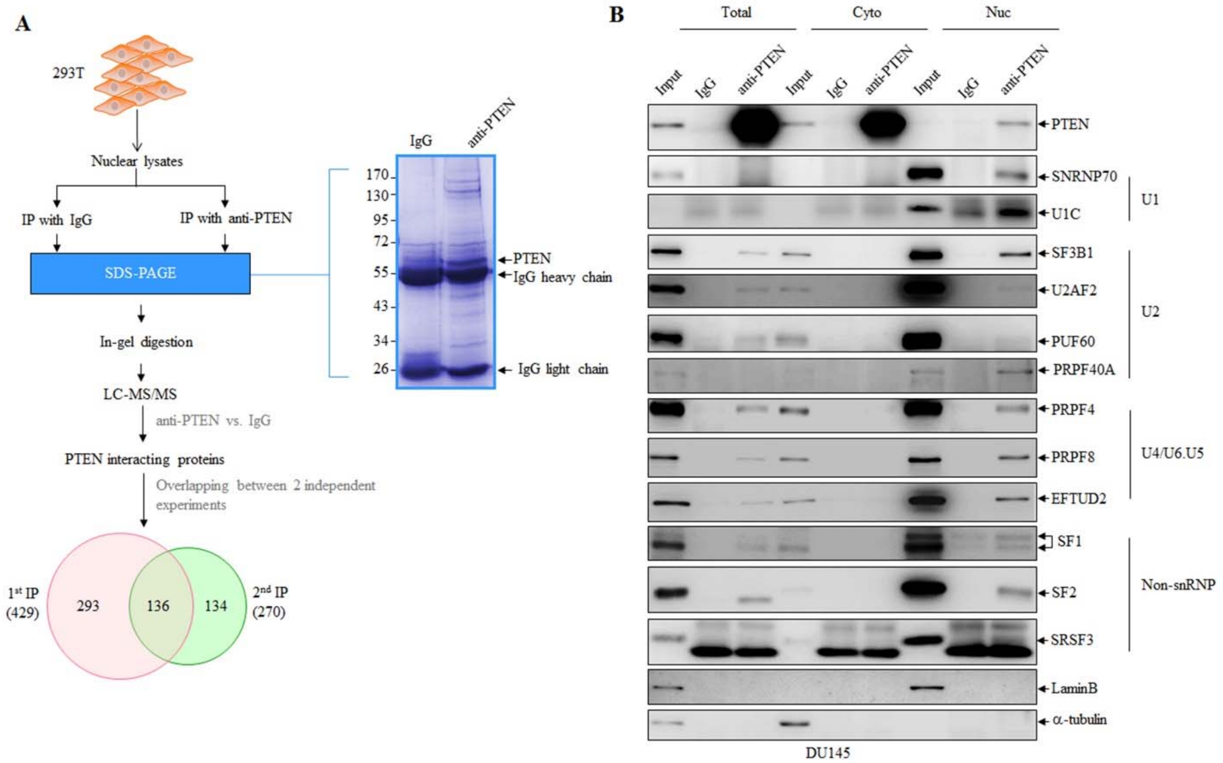

Supplementary Figure 3. Identification of nuclear PTEN interactome. (**A**) Experimental strategy and workflow for identification of PTEN-interacting proteins in the nuclei. The numbers of proteins identified in two independent experiments were shown. (**B**) DU145 cells were fractionated into total, cytoplasmic and nuclear fractions, and immunoprecipitation with anti-PTEN antibody or control IgG was performed in each fraction, followed by immunoblotting for proteins as indicated. LaminB and α-tubulin serve as nuclear and cytoplasmic markers respectively.

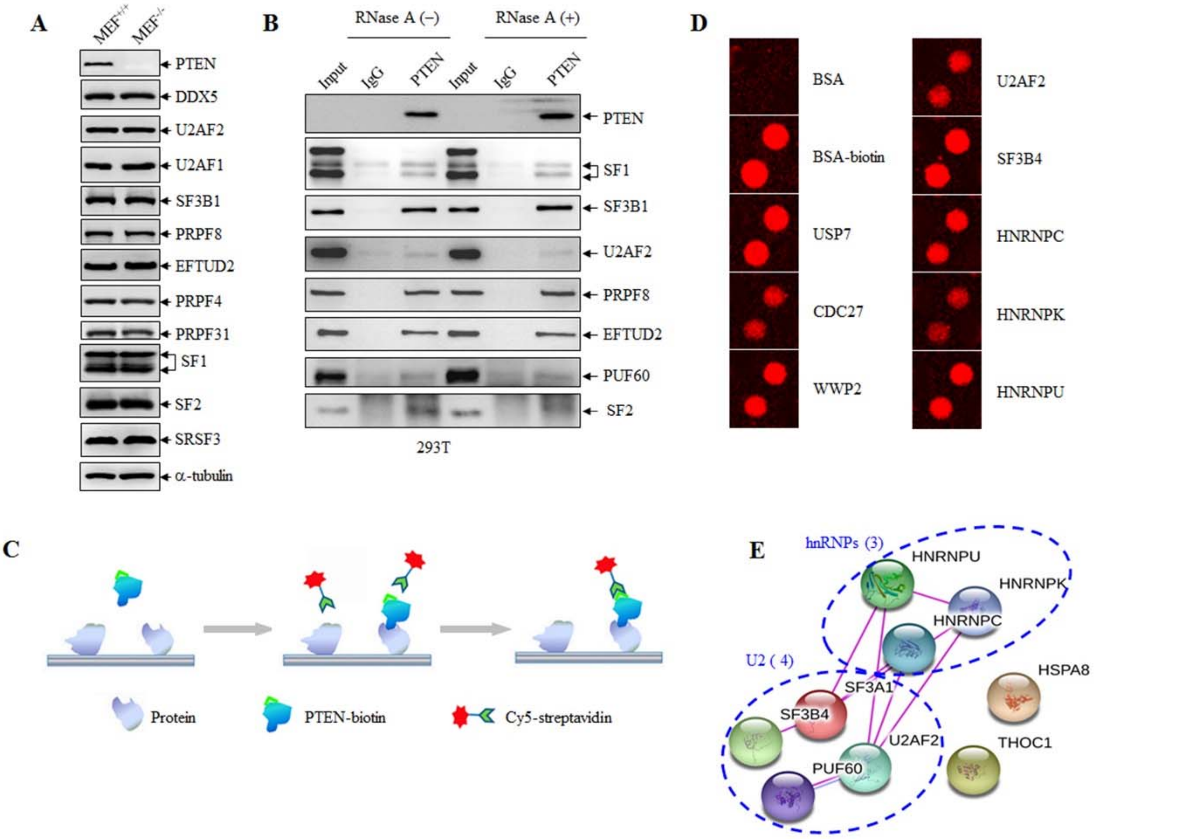

Supplementary Figure 4. Identification of proteins directly interacting with PTEN. (**A**) Immunoblotting of proteins as indicated in PTEN$^{+/+}$ and PTEN$^{-/-}$ MEF cells. (**B**) Lysates of 293T nuclear fractions treated with or without 100 μg/ml RNase for 1 h at room temperature were immunoprecipitated with anti-PTEN antibody or control IgG, followed by immunoblotting for indicated proteins. (**C**) Workflow of the human proteome microarray strategy for the identification of direct protein-protein interactions. (**D**) Representative PTEN-interacting proteins, with bovine serum albumin (BSA) and BSA-biotin serving as negative and positive controls respectively. PTEN–biotin conjugate did not bind BSA, suggesting the specificity of the assay. (**E**) Analysis of the PTEN-interacting spliceosomal proteins by String database. The edge indicates known interaction between two proteins. Proteins in the same complex or family were circled by the broken line.

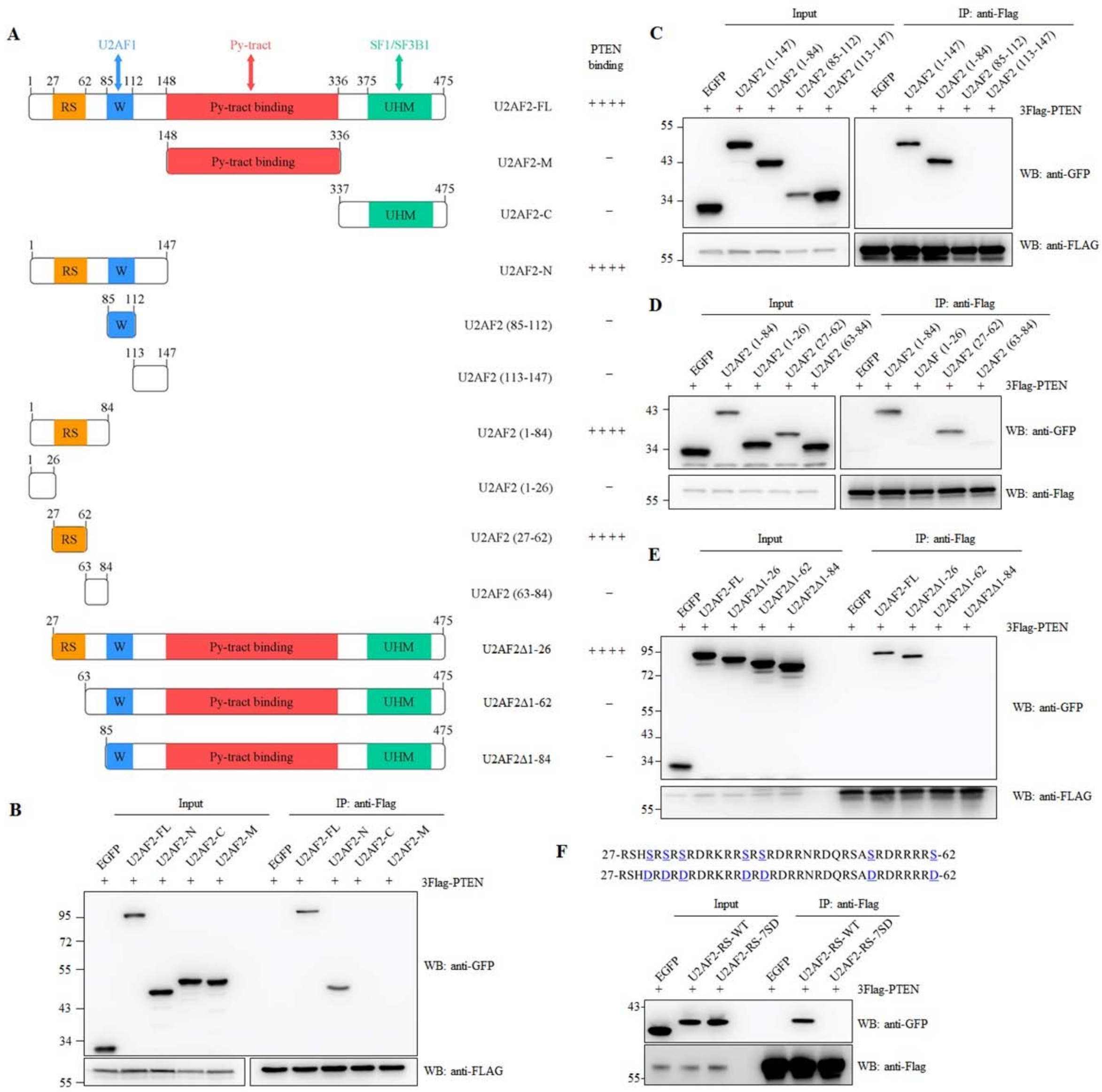

Supplementary Figure 5. Mapping the domain of U2AF2 interacting PTEN. (**A**) The domain structures of U2AF2 and deletion mutants. The PTEN-binding strength of each construct is shown on the right, with "+" representing one unit of binding strength, and "–" representing no binding. (**B-E**) 3×Flag-tagged PTEN and EGFP-tagged U2AF2 as indicated in (A) were co-transfected into 293T cells, and immunoprecipitation with anti-Flag antibody was performed, followed by immunoblotting by by anti-Flag or GFP antibody. (**F**) A phosphomimetic mutant of U2AF2 RS domain was constructed by substituting seven indicated serines (S) with aspartic acids (D) (top). EGFP-tagged U2AF2 RS domain, the phosphomimetic mutant along with EGFP were co-transfected with 3×Flag-tagged PTEN into 293T cells, and immunoprecipites with anti-Flag antibody were immunobloted by anti-Flag or GFP antibody.

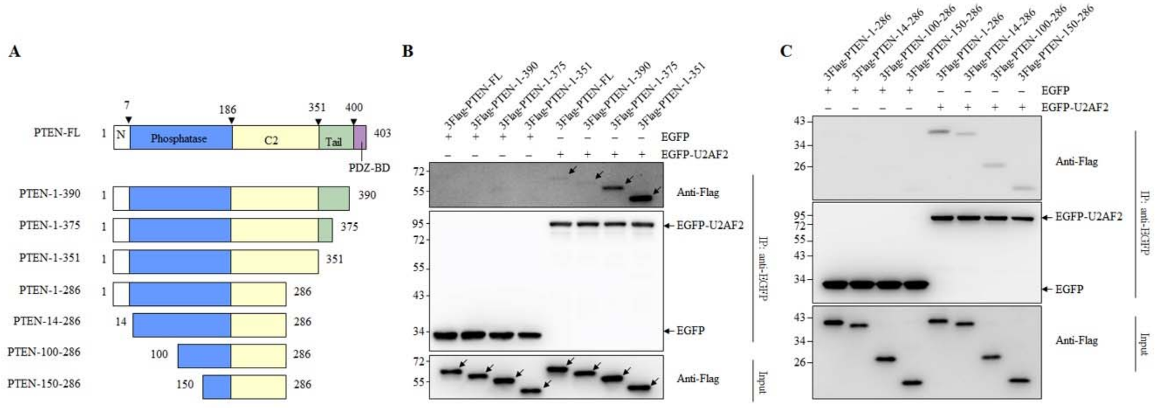

Supplementary Figure 6. Mapping the domain of PTEN interacting with U2AF2. (**A**) The domain structures of PTEN and its deletion mutants. (**B**, **C**) EGFP or EGFP-tagged U2AF2 was co-transfected with 3×Flag-tagged PTEN into 293T cells, and immunoprecipitation with anti-EGFP antibody was performed, followed by immunoblotting for 3×Flag- and GFP-tagged proteins.

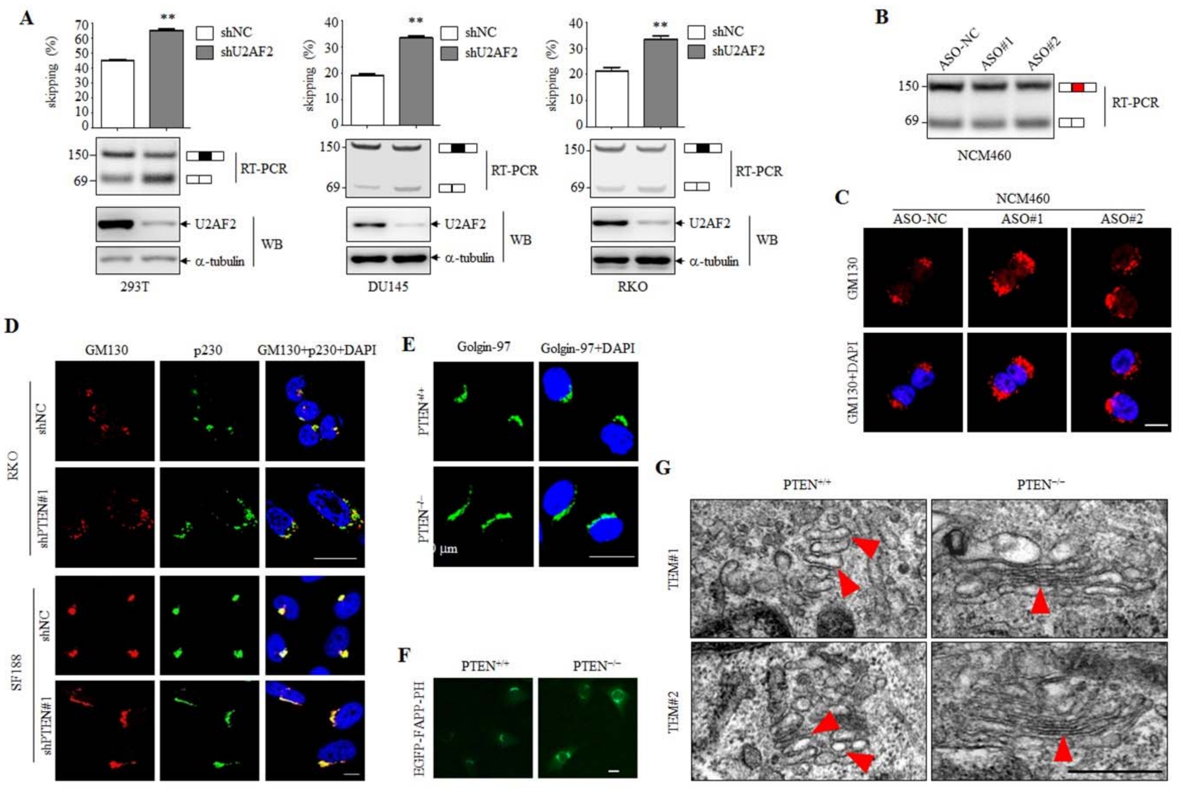

Supplementary Figure 7. PTEN loss promotes GOLGA2 skipping and Golgi extension. (**A**) Analysis of GOLGA2 skipping by U2AF2 knockdown. A representative example of RT-PCR analysis (middle) and immunoblotting for indicated proteins (bottom) and quantification of three independent biological replicates (top) are shown. Data represent means with bar as s.d. of three independent experiments. **, $p < 0.01$; two-sided unpaired *t*-test. (**B**) RT-PCR verification of GOLGA2 skipping in ASOs-transfected NCM460 cells. (**C**) Immunofluorescent staining of GM130 together with re-staining of DAPI in ASOs-transfected NCM460 cells. Scale bar represents 10 μm. (**D**) Immunofluorescent staining of GM130 and p230 together with re-staining of DAPI in RKO and SF188 cells with or without PTEN knockdown. Scale bar represents 20 μm and 10 μm for RKO and SF188 cells respectively. (**E**) Immunofluorescent staining of Golgin-97 together with re-staining of DAPI in PTEN$^{+/+}$ and PTEN$^{-/-}$ cells. Scale bar represents 20 μm. (**F**) PTEN$^{+/+}$ and PTEN$^{-/-}$ MEF cells were transfected with EGFP-tagged PH domain from FAPP1 and observed with fluorescence microscope. Scale bar represents 20 μm. (**G**) Golgi structure was analyzed in TEM images of PTEN$^{+/+}$ and PTEN$^{-/-}$ MEF cells. Two fields are shown for each cell. The red triangles point to Golgi. Scale bar represents 500 nm.

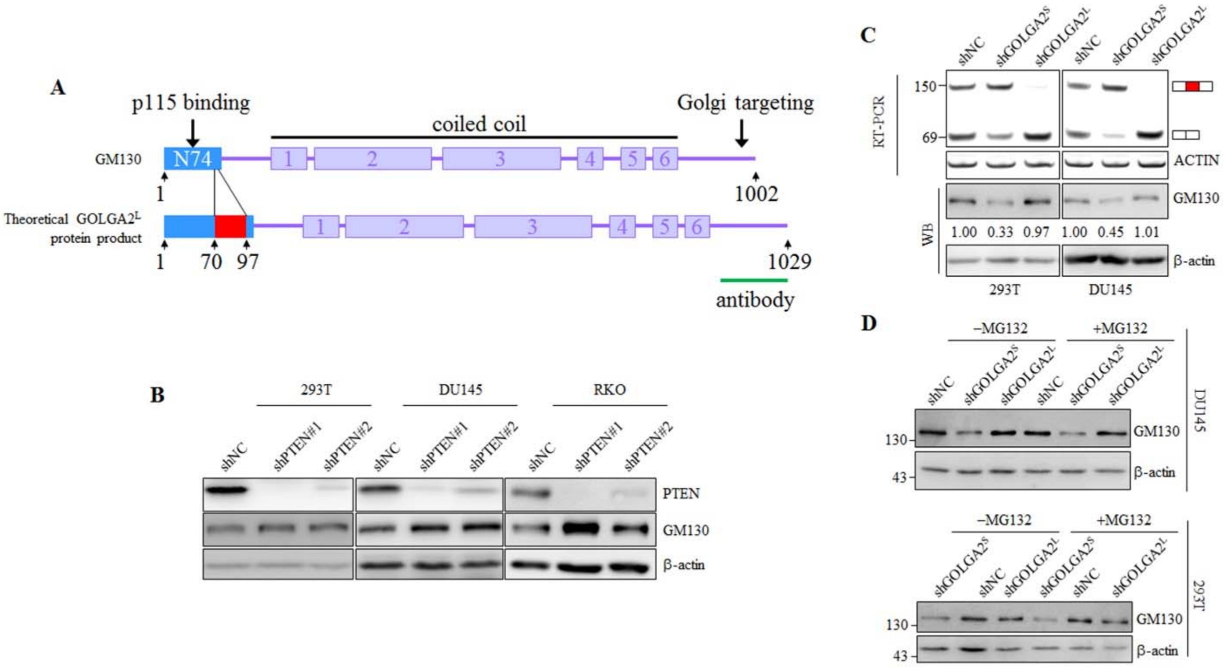

Supplementary Figure 8. PTEN deficiency promotes GM130 expression. (**A**) The domain structures of GM130 encoded by GOLGA2^{S} and and the theoretical protein product of GOLGA2^{L} are shown. The red rectangle indicates amino acids encoded by exon 2b. (**B**) Immunoblotting of indicated proteins in 293T, DU145 and RKO cells with or without PTEN knockdown. (**C**) RT-PCR analysis of GOLGA2^{S} and GOLGA2^{L} isoforms in 293T and DU145 cells infected with shRNAs designed to target GOLGA2^{S} or GOLGA2^{L} (top). Immunoblotting for indicated proteins are shown in the bottom panel. GM130 was quantified and normalized to β-actin. (**D**) The same cell lines in (C) were treated with or without MG132 for 8 h. Immunoblotting for indicated proteins are shown.

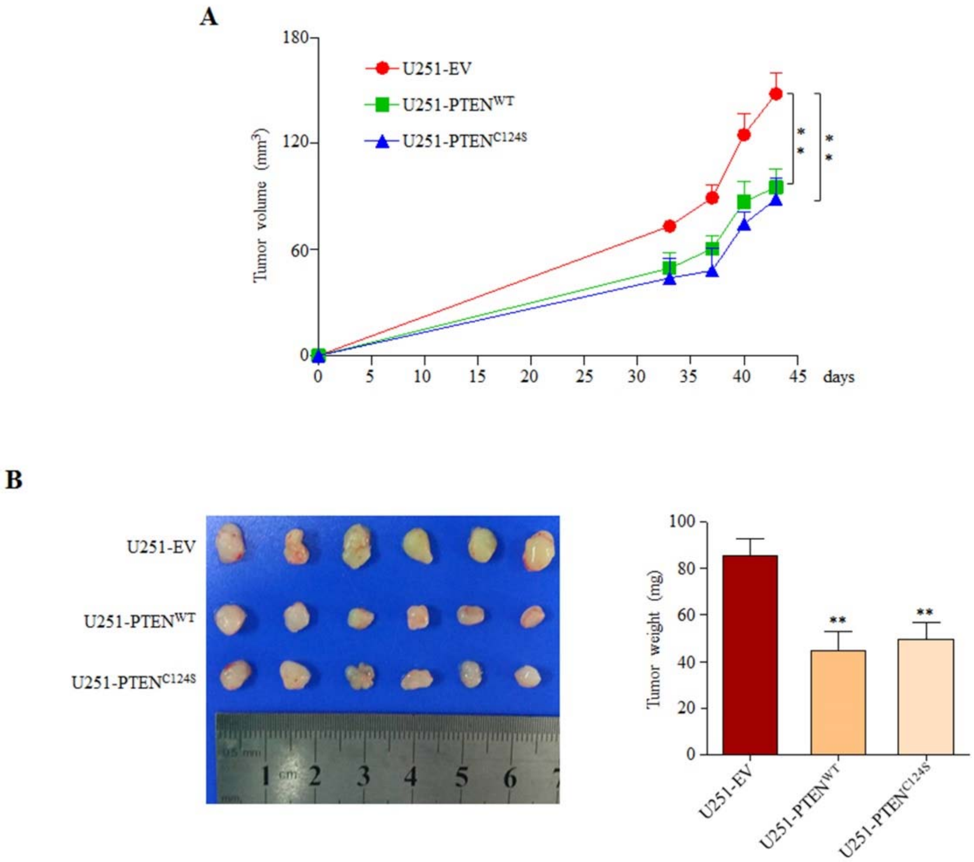

Supplementary Figure 9. PTEN^{WT} and PTEN^{C124S} show similar tumor suppressive capacities in U251 cells. (**A**, **B**) U251 cells were infected with PTEN^{WT} or PTEN^{C124S} along with EV viruses, and subcutaneously injected into nude mice. Tumor volumes were measured at different time points (A). Forty-three days after subcutaneous injection, the tumors were harvested and weighed (B). Data represent means with bar as s.d..**, $p < 0.01$; two-sided paired *t*-test for (A); two-sided unpaired *t*-test for (B).

Fig.1b

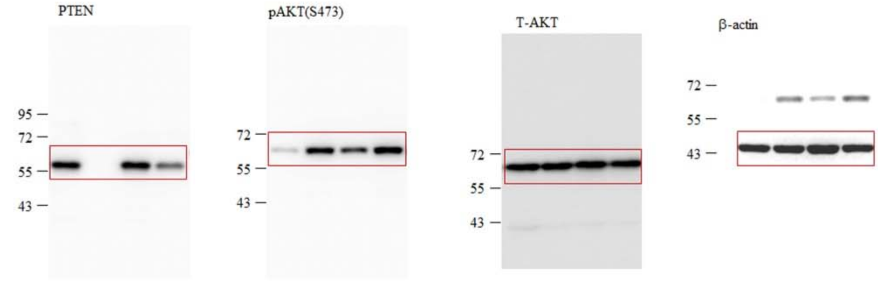
PTEN
95
72
55
43
pAKT(S473)
72
55
43
T-AKT
72
55
43
β-actin
72
55
43

Fig.3d

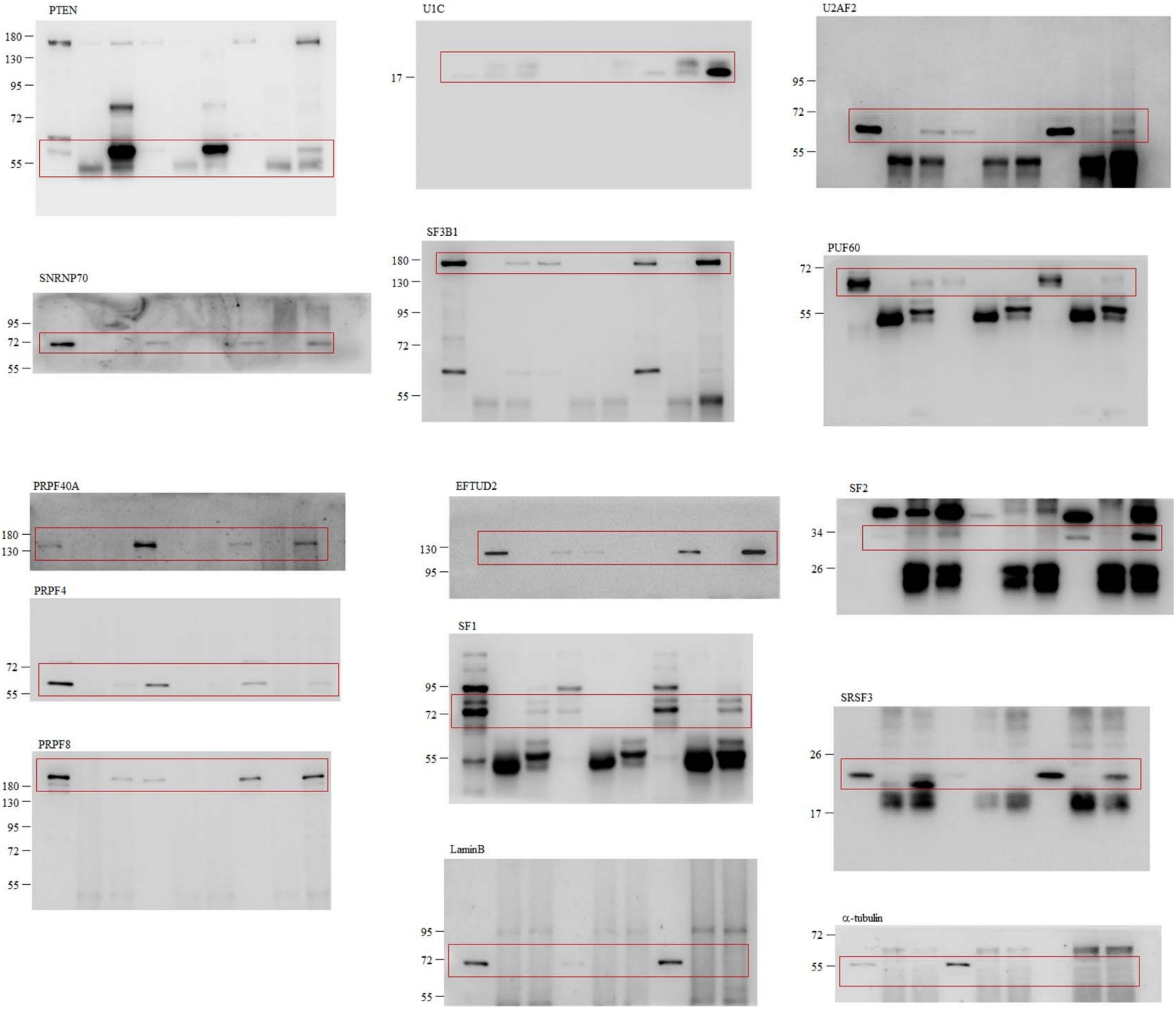
PTEN
180
130
95
72
55
U1C
17
U2AF2
95
72
55
SNRNP70
95
72
55
SF3B1
180
130
95
72
55
PUF60
72
55
PRPF40A
180
130
EFTUD2
130
95
SF2
34
26
PRPF4
72
55
SF1
95
72
55
SRSF3
26
17
PRPF8
180
130
95
72
55
LaminB
95
72
55
α-tubulin
72
55

Fig. 3e

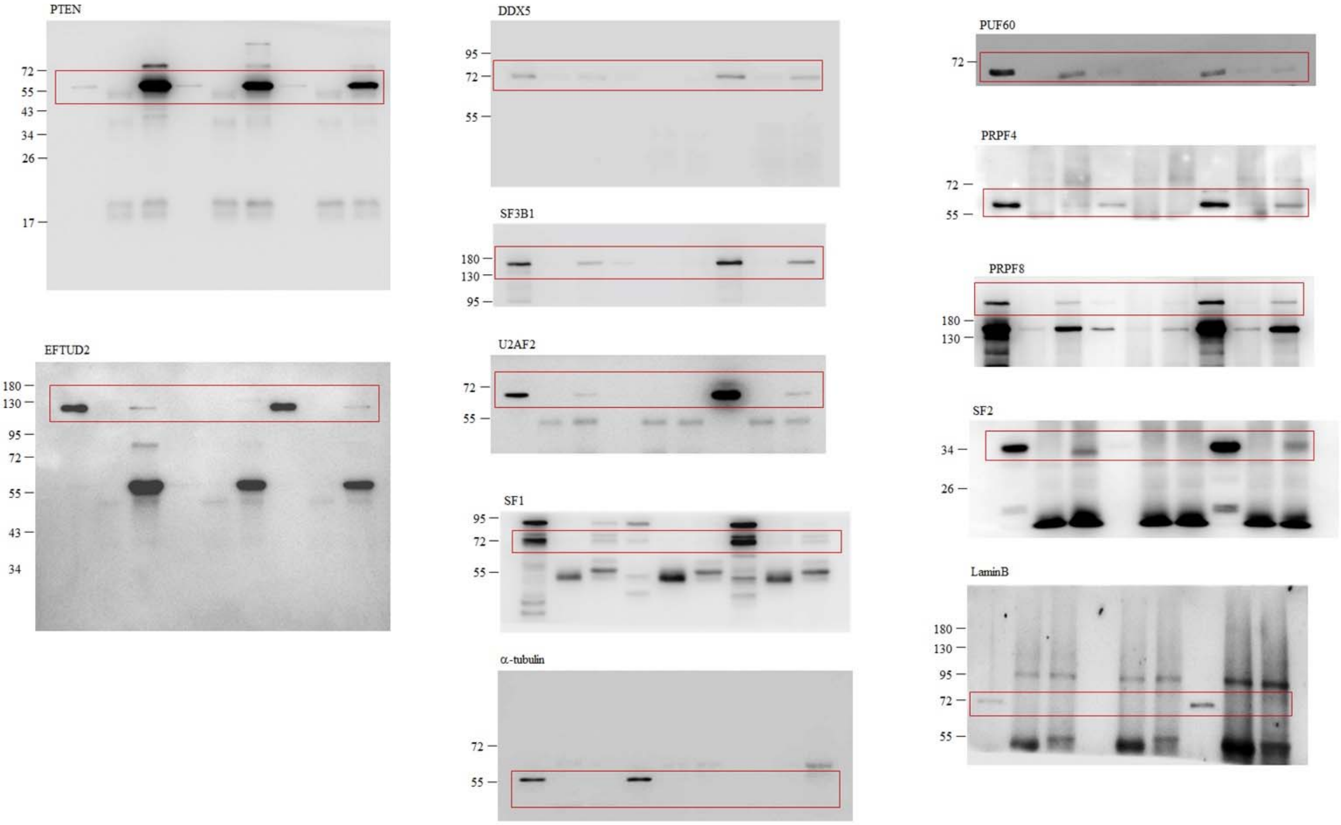

Supplementary Fig. 3B

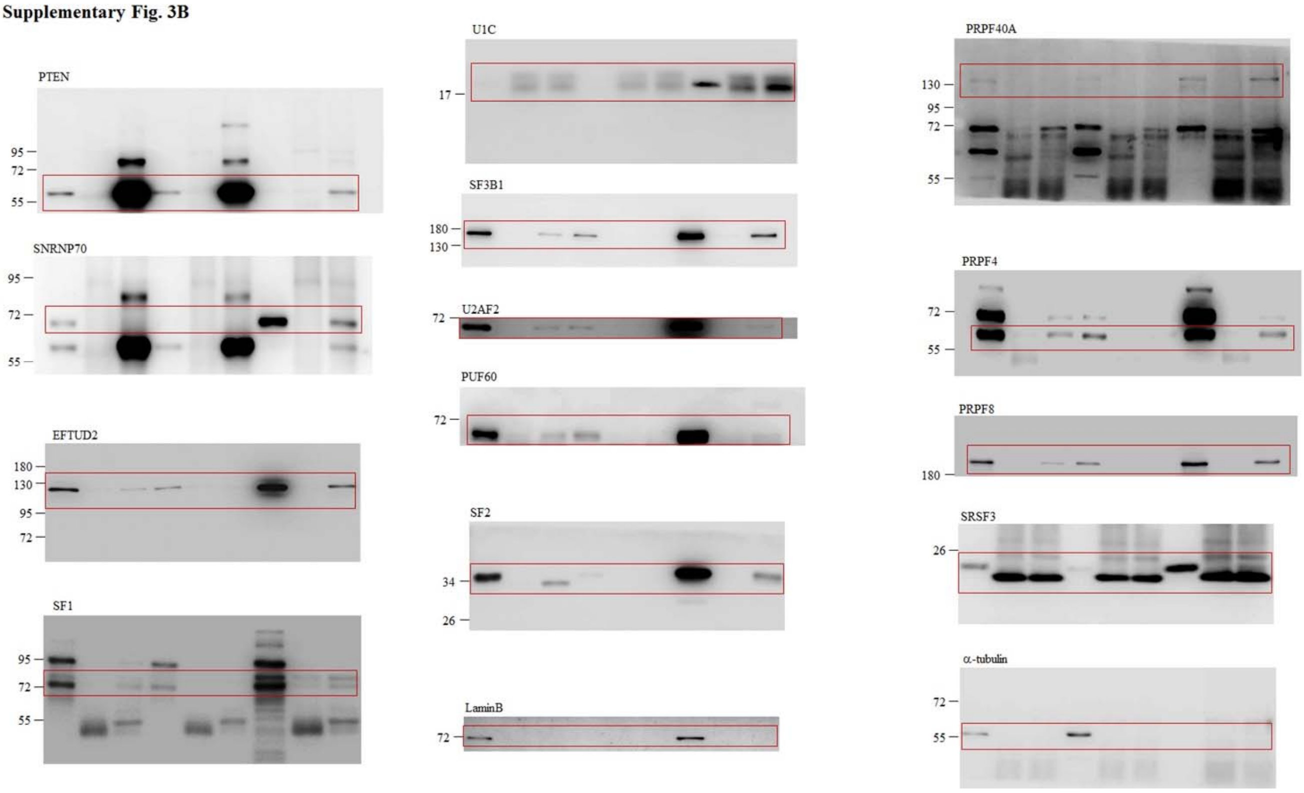

Fig. 4e

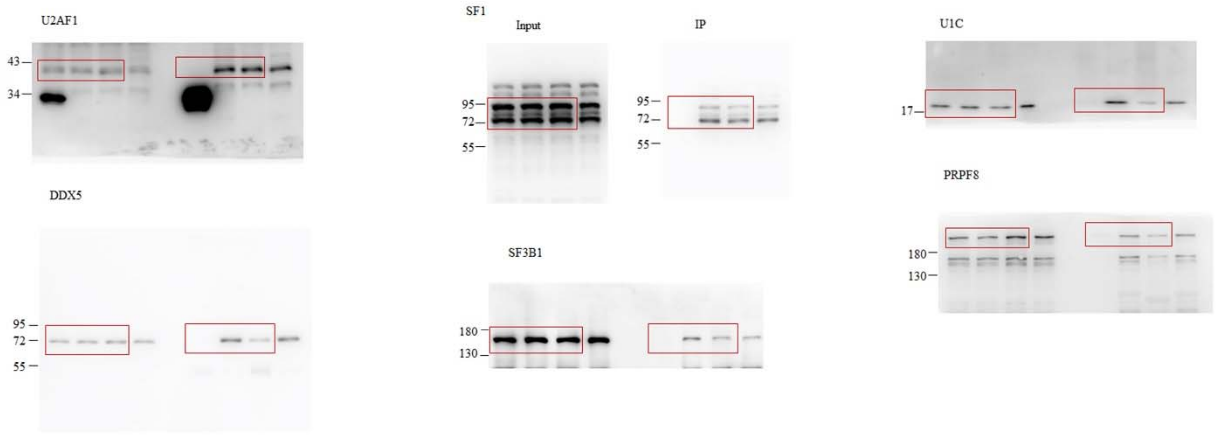
U2AF1
43
34
SF1
Input
IP
95
72
55
U1C
17
PRPF8
180
130
DDX5
95
72
55
SF3B1
180
130

Fig. 4f

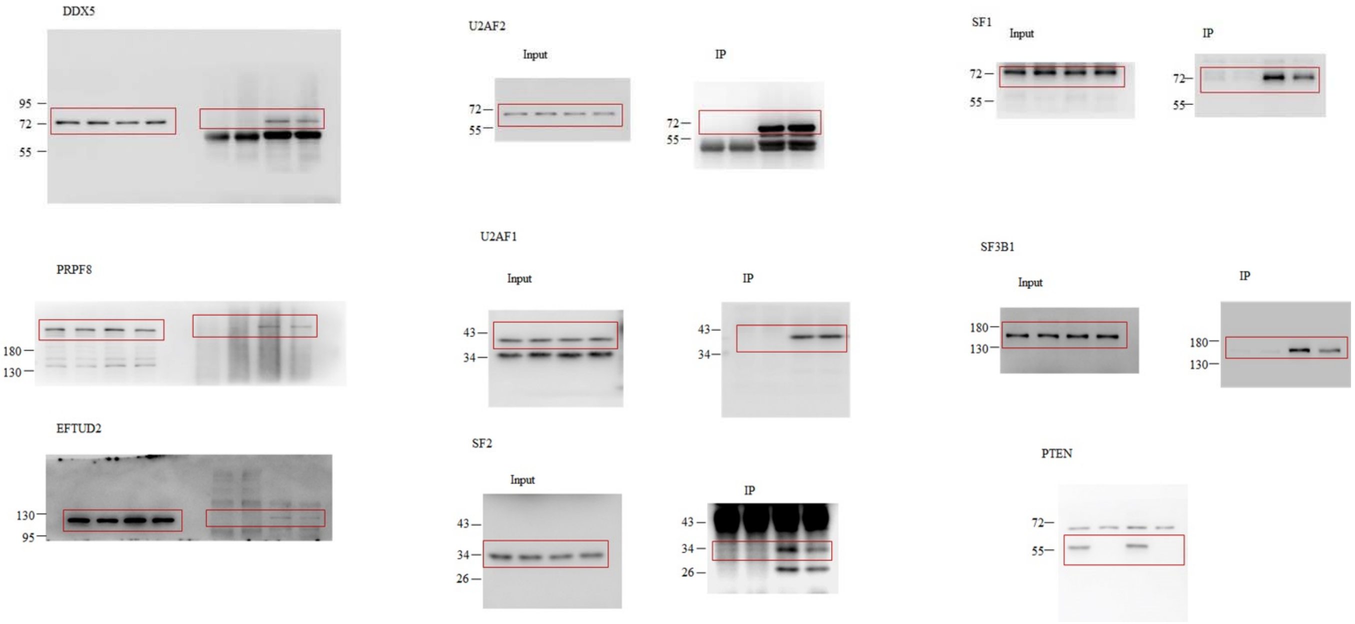
DDX5
95
72
55
U2AF2
Input
IP
72
55
SF1
Input
IP
72
55
PRPF8
180
130
U2AF1
Input
IP
43
34
SF3B1
Input
IP
180
130
EFTUD2
130
95
SF2
Input
IP
43
34
26
PTEN
72
55

Fig. 4g

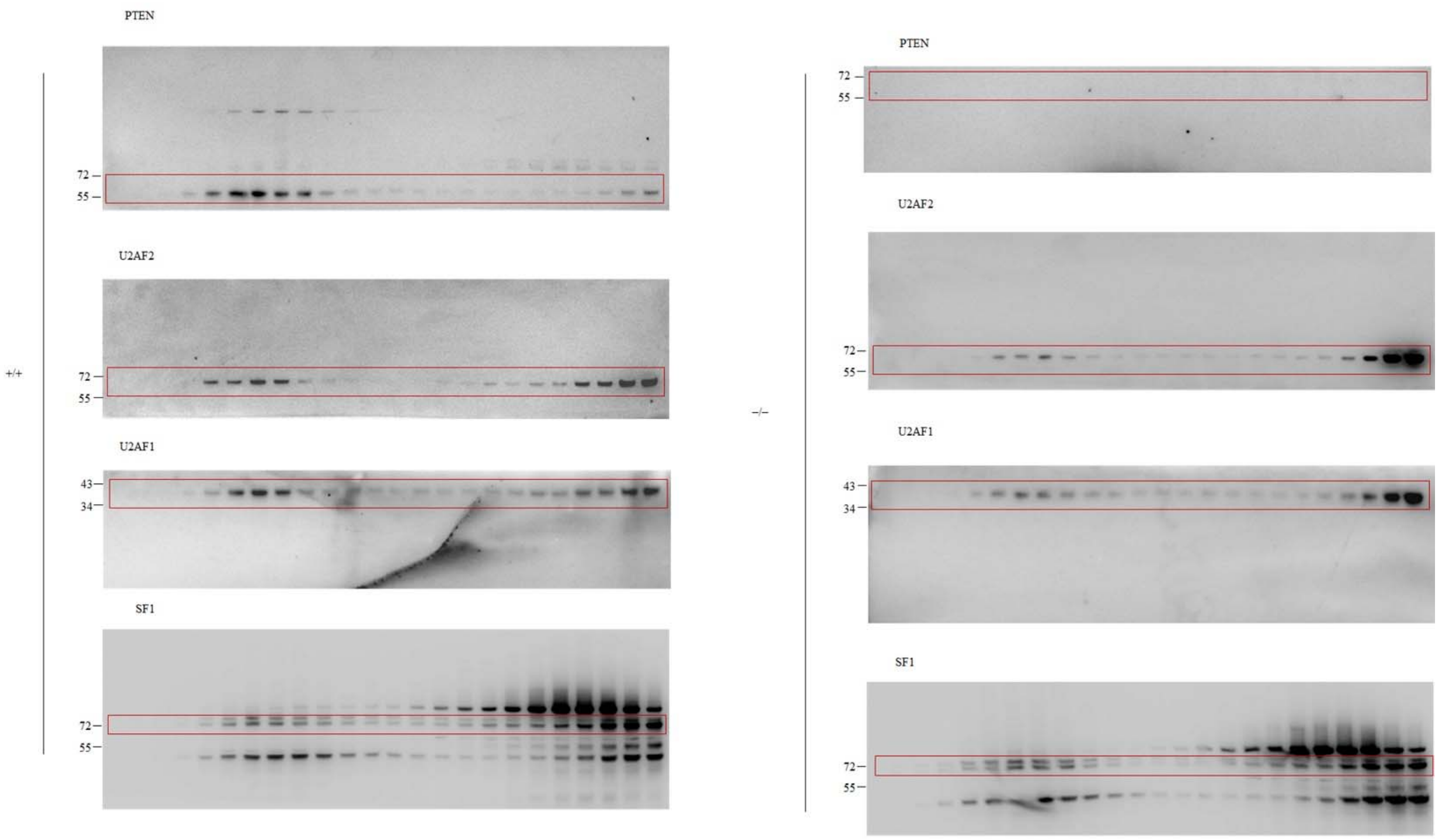

Fig. 7e

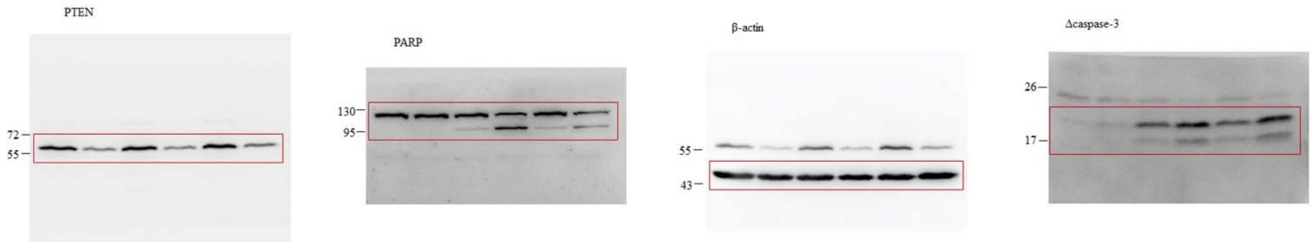

Supplementary Figure 10. Uncropped images of immunoblots. Uncropped images of several important immunoblots displayed in the main figures of the manuscript. Molecular weight markers displayed on the left side. Red boxes mark the relevant protein.

Supplementary Table 1. PTEN-regulated ASEs significantly correlated with the status of PTEN copy number in GBMLGG.

**genename_ASE_ID**	**GeneName**	**p value (-log10)**	**ID**
MAGI3	MAGI3	6.32	chr1:114223958:+,chr1:114224834:+@chr1:114223958:+,chr1:114225519:+
PRUNE	PRUNE	2.20	chr1:150999803:+,chr1:151001262:+@chr1:150999803:+,chr1:151006282:+
LPHN2	LPHN2	27.76	chr1:82417826:+,chr1:82418671:+@chr1:82417826:+,chr1:82421561:+
PTBP2	PTBP2	26.00	chr1:97270495:+,chr1:97271975:+@chr1:97270495:+,chr1:97272422:+
ARHGAP12	ARHGAP12	4.12	chr10:32128247:-,chr10:32128565:-@chr10:32120728:-,chr10:32128565:-
ARHGEF12_1	ARHGEF12	3.57	chr11:120278532:+,chr11:120280103:+@chr11:120278532:+,chr11:120291462:+
ARHGEF12_2	ARHGEF12	2.14	chr11:120280159:+,chr11:120291462:+@chr11:120278532:+,chr11:120291462:+
C2CD5	C2CD5	4.57	chr12:22612476:-,chr12:22622643:-@chr12:22610095:-,chr12:22622643:-
KIF21A	KIF21A	11.72	chr12:39724064:-,chr12:39724548:-@chr12:39720126:-,chr12:39724548:-
RBM26	RBM26	4.49	chr13:79918929:-,chr13:79927288:-@chr13:79918929:-,chr13:79928574:-
ANKRD12	ANKRD12	7.74	chr18:9195696:+,chr18:9204474:+@chr18:9195696:+,chr18:9208655:+
MFF_2	MFF	2.43	chr2:228205096:+,chr2:228211942:+@chr2:228205096:+,chr2:228220393:+
MFF_1	MFF	2.46	chr2:228217289:+,chr2:228220393:+@chr2:228205096:+,chr2:228220393:+
ATG16L1	ATG16L1	18.79	chr2:234181698:+,chr2:234182367:+@chr2:234181698:+,chr2:234183322:+
FAM161A	FAM161A	1.83	chr2:62063244:-,chr2:62066556:-@chr2:62065840:-,chr2:62066556:-
CD47	CD47	3.78	chr3:107768498:-,chr3:107769425:-@chr3:107766139:-,chr3:107769425:-
LRCH3	LRCH3	3.83	chr3:197581316:+,chr3:197585705:+@chr3:197581316:+,chr3:197592294:+
ARMC10	ARMC10	2.20	chr7:102727211:+,chr7:102732924:+@chr7:102727211:+,chr7:102737724:+
GOLGA2	GOLGA2	16.65	chr9:131035144:-,chr9:131036129:-@chr9:131030803:-,chr9:131036129:-
OCRL	OCRL	10.60	chrX:128710529:+,chrX:128718321:+@chrX:128710529:+,chrX:128720979:+

Table column explanation: genenames_ASE_ID, most of them are official gene symbols. If a gene have multiple ASEs in this table, it will be denoted as GenName_ID, such as ARHGEF12_1; GeneName, official gene symbols; Fisher test's pvalue, the p value of Fisher exact test; ID, it is constructed from both skipping junction (before the symbol @) and inclusion junction (after the symbol @).

Supplementary Table 2. PTEN-regulated ASEs correlated with patient survival in GBMLGG.

Genename ASE ID	genename	P_value	Hazard Ratio	CI95 (lower)	CI95 (upper)	ID
MFF_1	MFF	0.048	1.52	1.00	2.31	chr2:228217289:+,chr2:228220393:+@chr2:228205096:+,chr2:228220393:+
SNX14	SNX14	0.046	2.74	1.02	7.35	chr6:86248582:-,chr6:86251703:-@chr6:86246642:-,chr6:86251703:-
CACNA1H	CACNA1H	0.039	0.73	0.54	0.98	chr16:1262138:+,chr16:1262511:+@chr16:1262138:+,chr16:1263780:+
ADD3	ADD3	0.026	2.51	1.12	5.64	chr10:111890244:+,chr10:111893084:+@chr10:111890244:+,chr10:111892063:+
FAM161A	FAM161A	0.012	2.46	1.22	4.99	chr2:62063244:-,chr2:62066556:-@chr2:62065840:-,chr2:62066556:-
MFF_2	MFF	0.0092	1.58	1.12	2.23	chr2:228205096:+,chr2:228211942:+@chr2:228205096:+,chr2:228220393:+
ARMC10	ARMC10	0.0063	2.67	1.32	5.41	chr7:102727211:+,chr7:102732924:+@chr7:102727211:+,chr7:102737724:+
EXOC1	EXOC1	0.0036	4.37	1.62	11.81	chr4:56750094:+,chr4:56756389:+@chr4:56750094:+,chr4:56755054:+
ARHGAP12	ARHGAP12	0.0030	2.14	1.29	3.54	chr10:32128247:-,chr10:32128565:-@chr10:32120728:-,chr10:32128565:-
PARP11	PARP11	0.00051	0.54	0.38	0.76	chr12:3935399:-,chr12:3939056:-@chr12:3938196:-,chr12:3939056:-
CD47	CD47	0.00016	2.06	1.42	3.00	chr3:107768498:-,chr3:107769425:-@chr3:107766139:-,chr3:107769425:-
RBM26	RBM26	5.76E-06	1.60	1.30	1.95	chr13:79918929:-,chr13:79927288:-@chr13:79918929:-,chr13:79928574:-
OCRL	OCRL	1.70E-06	1.88	1.45	2.44	chrX:128710529:+,chrX:128718321:+@chrX:128710529:+,chrX:128720979:+
KIF21A	KIF21A	1.38E-06	2.04	1.53	2.73	chr12:39724064:-,chr12:39724548:-@chr12:39720126:-,chr12:39724548:-
MAGI3	MAGI3	5.71E-07	2.70	1.83	3.99	chr1:114223958:+,chr1:114224834:+@chr1:114223958:+,chr1:114225519:+
C2CD5	C2CD5	8.56E-08	2.18	1.64	2.90	chr12:22612476:-,chr12:22622643:-@chr12:22610095:-,chr12:22622643:-
LPHN2	LPHN2	5.85E-08	2.00	1.56	2.57	chr1:82417826:+,chr1:82418671:+@chr1:82417826:+,chr1:82421561:+
PTBP2	PTBP2	1.83E-08	1.96	1.55	2.48	chr1:97270495:+,chr1:97271975:+@chr1:97270495:+,chr1:97272422:+
GOLGA2	GOLGA2	1.30E-08	1.78	1.46	2.16	chr9:131035144:-,chr9:131036129:-@chr9:131030803:-,chr9:131036129:-
ANKRD12	ANKRD12	6.21E-09	2.33	1.75	3.10	chr18:9195696:+,chr18:9204474:+@chr18:9195696:+,chr18:9208655:+
ATG16L1	ATG16L1	1.99E-10	2.04	1.64	2.55	chr2:234181698:+,chr2:234182367:+@chr2:234181698:+,chr2:234183322:+

Table column explanation: genenames_ASE_ID, most of them are official gene symbols. If a gene have multiple ASEs in this table, it will be denoted as GenName_ID, such as MFF_2; GeneName, official gene symbols; P_value, the p value of log rank test; HazardRatio, the hazard ratio value; CI95_lower, the lower value of 95% confidence interval of hazard ratio; CI95_upper, the upper value of 95% confidence interval of hazard ratio; ID, it is constructed from both skipping junction (before the symbol @) and inclusion junction (after the symbol @).

Supplementary Table 3. Antibody information.

Antigen	Manufacturer	Catalog no	Dilution
**WB**			
AKT	Cell Signaling Technology	4691	1:1000
AKT (S473)	Cell Signaling Technology	4060s	1:1000
DDX5	Abcam	ab126730	1:1000
EFTUD2	Abcam	ab188327	1:1000
FLAG	Sigma	F1804	1:1000
GFP	Abcam	ab183734	1:1000
GM130	Proteintech Group	11308-1-AP	1:1000
GST	Cell Signaling Technology	2625s	1:1000
HIS	Beyotime	AH367	1:1000
PTEN	Cell Signaling Technology	9559L	1:1000
PRPF4	Abcam	ab198998	1:1000
PRPF40A	Abcam	ab204371	1:1000
LaminB	Santa Cruz	sc-6216	1:1000
PUF60	Abcam	ab184538	1:1000
SF1	Abcam	ab58077	1:1000
SF2	Abcam	ab129108	1:1000
SF3b1	Abcam	ab172634	1:1000
SNRNP70	Abcam	Ab83306	1:1000
SRSF3	Abcam	ab125124	1:1000
U1C	Abcam	ab157116	1:1000
U2AF35	Abcam	ab197591	1:1000
U2AF65	Abcam	ab197031	1:1000
α-tubulin	MBL	PM054-7	1:1000
β-actin	MBL	PM053-7	1:1000
PRPF8	Abcam	ab185547	1:1000
**IP**			
PTEN (IP)	Cell Signaling Technology	9556S	1:200
U2AF65	Abcam	ab197031	1:200
**IF**			
GM130 (IF)	Abcam	ab52649	1:100
p230	BD Transduction Laboratories	611280	1:100
Golgin-97	Cell Signaling Technology	13192s	1:100
VSVG	Kerafast	8G5F11	1:100

Supplementary Table 4. Primer information.

Quantitative real-time PCR primers			
	snRNA	Forward primer (5'->3')	Reverse primer (5'->3')
mouse	U1	ctgaggcatagtggctatgga	ggatctccctgtcttttgtttgt
	U2	tgttgagtgtatggggcgtg	gaccattcttattccccagaggg
	U4	gctttgcgcagtggcagtat	tctccgtagagactgtcaaaaattg
	U5	ctggtttctcttcagatcgt	gttggagcagaacctcaaaaaatt
	U6	gctcgcttcggcagcacata	aatatggaacgcttcacgaat
RT-PCR primers			
	GN	Forward primer (5'->3')	Reverse primer (5'->3')
human	*GOLGA2*	ccacttctggtggttgccac	cagatggttgtagagtagca
	UAP	agatgaaaatggctctcgccttc	ggattggtacatcattggcatcc
	SNX14	aggtgcagaatcaccaacacg	gctgattccaaatgattctcccc
	APP	gaagagtactgcatggccgt	gatacttgtcaacggcatcagg
	CD46	cccgtacagatatcttcaaaggag	taagtggcatattcagctccacc
	SPAG9	ggaaatggtgtcattatctccatcc	aggacgatttcctggtacacctg
	KIF21A	gctagatgctttactaggccat	cctcatcagtactatcctctac
	C2CD5	tcacttccttctcatccttttcca	gacttgctttttcaactgtcatgg
mouse	*golga2*	ccactgccagtgactgtcag	gtgcaggagcaatatggtct

Primers for PTEN synonymous mutation	
Forward primer (5'->3')	Reverse primer (5'->3')
gacctcgaccagtggctgagtgaagatga	tcatcttcactcagccactggtcgaggtc
