# Supplementary material for: Nuclear PTEN safeguards pre-mRNA splicing to link Golgi apparatus for its tumor suppressive role
Source: Nat Commun. 2018 Jun 19;9:2392. doi: 10.1038/s41467-018-04760-1 (PMC6008332; doi:10.1038/s41467-018-04760-1)
Supplement: Supplementary file 3 — Description of Additional Supplementary Files [file 41467_2018_4760_MOESM3_ESM.pdf]

Description of Additional Supplementary Files

File Name: Supplementary Data 1
Description: 262 common PTEN-regulated ASEs were identified from shRNA#1 and shRNA#2 293T cells.

File Name: Supplementary Data 2
Description: Tumour prevalence of PTEN-regulated ASEs identified in 293T cells in TCGA datasets.

File Name: Supplementary Data 3
Description: Splicing ratio values of skipping junctions of ASEs induced by shPTEN#1 were increased by the ectopic expression of PTEN-WT and PTEN-C124S.

File Name: Supplementary Data 4
Description: co-IP LC-MS/MS identification results in two independent assays.

File Name: Supplementary Data 5
Description: PTEN probed HPM data.
